# Heterologous Prime-Boost Combinations Highlight the Crucial Role of Adjuvant in Priming the Immune System

**DOI:** 10.3389/fimmu.2018.00380

**Published:** 2018-03-12

**Authors:** Annalisa Ciabattini, Elena Pettini, Fabio Fiorino, Simone Lucchesi, Gabiria Pastore, Jlenia Brunetti, Francesco Santoro, Peter Andersen, Luisa Bracci, Gianni Pozzi, Donata Medaglini

**Affiliations:** ^1^Laboratory of Molecular Microbiology and Biotechnology, Department of Medical Biotechnologies, University of Siena, Siena, Italy; ^2^U&E PreMed Laboratory, Department of Medical Biotechnologies, University of Siena, Siena, Italy; ^3^Department of Infectious Disease Immunology, Statens Serum Institute, Copenhagen, Denmark

**Keywords:** prime-boost regimens, adjuvants, computational flow cytometry, T cells, intracellular cytokines, CAF01, priming

## Abstract

The induction and modulation of the immune response to vaccination can be rationally designed by combining different vaccine formulations for priming and boosting. Here, we investigated the impact of heterologous prime-boost approaches on the vaccine-specific cellular and humoral responses specific for a mycobacterial vaccine antigen. C57BL/6 mice were primed with the chimeric vaccine antigen H56 administered alone or with the CAF01 adjuvant, and boosted with H56 alone, or combined with CAF01 or with the squalene-based oil-in-water emulsion adjuvant (o/w squalene). A strong secondary H56-specific CD4^+^ T cell response was recalled by all the booster vaccine formulations when mice had been primed with H56 and CAF01, but not with H56 alone. The polyfunctional nature of T helper cells was analyzed and visualized with the multidimensional flow cytometry FlowSOM software, implemented as a package of the R environment. A similar cytokine profile was detected in groups primed with H56 + CAF01 and boosted with or without adjuvant, except for some clusters of cells expressing high level of IL-17 together with TNF-α, IL-2, and IFN-γ, that were significantly upregulated only in groups boosted with the adjuvants. On the contrary, the comparison between groups primed with or without the adjuvant showed a completely different clusterization of cells, strengthening the impact of the formulation used for primary immunization on the profiling of responding cells. The presence of the CAF01 adjuvant in the priming formulation deeply affected also the secondary humoral response, especially in groups boosted with H56 alone or o/w squalene. In conclusion, the presence of CAF01 adjuvant in the primary immunization is crucial for promoting primary T and B cell responses that can be efficiently reactivated by booster immunization also performed with antigen alone.

## Introduction

A key aspect for the generation of efficacious vaccines is the optimization of vaccine schedules capable of eliciting the more adequate immune response for a specific pathogen, balancing between humoral and cell-mediated immunity. The design of prime-boost vaccine combinations based on the selection of the vaccine formulation, the dose, the route, and the intervals between doses is therefore of critical importance for optimally shaping the immune response.

With the exception of very few antigens, such as certain toxins, almost all the purified proteins used as vaccine antigens generally induce a modest antibody response with little or no T cell response (1). Adjuvants have proven to be key components in vaccines, providing danger signals and triggering a sufficient activation of the innate system. The presence of the adjuvant allows to enhance and appropriately skew the immune responses toward a vaccine antigen (1, 2) and promotes the induction of long-lived immunological memory and protection. Profiling the mode of action of different adjuvants is of critical importance for the rationale design of vaccination strategies (3–5). One of the immunological events that play a pivotal role in the generation of a vaccine-specific immune response is the primary activation of T helper cells, due to its close relationship with long-term humoral immunity and induction of protective antibodies (6). Antigen-specific T helper clonal expansion, differentiation, and dissemination toward distal sites are regulated by different factors, such as the route of the primary immunization, the dose of the antigen, and the vaccine formulations (7–15). We have demonstrated that an efficient antigen-specific CD4^+^ T cell priming, generating cells capable of responding to booster immunization, is preferentially elicited by the subcutaneous, and not by the nasal route of immunization (3). Nevertheless, the nasal route can be efficiently used for booster immunization, when a local effector cellular response is aimed, since it promotes the recruitment of activated T cells into the lungs (3). Mathematical models can be used as a tool to estimate *in vivo* the probability of antigen-specific CD4^+^ T cell expansion and dissemination upon immunization with adjuvanted vaccine formulations (16). Clonally expanded CD4^+^ T cells exert the effector function producing cytokines. On the basis of the simultaneous expression of specific pattern of cytokines, Th cells are classified into functionally defined effector subpopulations. This fate is strongly affected by factors such as the local pro-inflammatory environment, the dose and the route of the vaccine used, and the adjuvant included in the vaccine formulation (17, 18).

Since the priming event impacts the type and quality of the induced immune response, we have recently characterized the mode of action of four different adjuvants, alum, a squalene-based oil-in-water emulsion (structurally similar to the licensed MF59 adjuvant), CpG ODN1826 (19), and the liposome system CAF01 (20), after a single immunization (4). Comparative analysis showed that CAF01 and o/w squalene were the strongest adjuvants capable of activating cellular response, with a Th1/Th2 and Th1/Th17 profile, respectively. O/w squalene rapidly induced the release of antigen-specific IgG in serum while CAF01 stimulated the germinal center (GC) reaction within the draining lymph nodes. A strong GC reaction was also observed in the presence of alum, even if an early humoral response was not detected. On the contrary, CpG ODN adjuvant elicited a rapid humoral response, but not a CD4^+^ T cell activation and only a mild GC reaction, suggesting a T-independent activation of the B cell response, due to the direct stimulation of TLRs on B cells (21). With these information, rationale combination of adjuvants can be exploited for designing vaccination approaches capable of eliciting the most adequate immune response for a specific pathogen. The strategy of generating a toolbox of adjuvants, with a well-defined profile to shape the immune response, has also been recently identified as a key priority in vaccine research and development in Europe^1^ (22).

When many parameters are combined in a flow cytometric analysis for studying the phenotype, the effector function, and the polyfunctionality of activated cells, as is the case of the characterization of an immune response elicited by vaccination, classical two-dimensional scatter plots analysis cannot be sufficient for the multidimensional nature of the data. To overcome this problem, novel computational techniques have been developed in the recent years, and computational flow cytometry has become a novel discipline useful for providing a set of tools to analyze, visualize, and interpret large amounts of cell data in a more automated and unbiased way (23). FlowSOM is an advanced visualization technique in which more information are provided than in the traditional two-dimensional scatter plots (24). A self-organizing map (SOM) is an unsupervised technique for clustering and dimensionality reduction, in which a discretized representation of the input space is trained. With FlowSOM, cells are grouped into cell type clusters that are then represented in a lower-dimensional space. This approach allows to visualize in the same picture information regarding the frequency of cells co-expressing different markers, and to compare different groups.

In this work, we have assessed different prime-boost combinations, using the CAF01 and o/w squalene adjuvants, in order to dissect their role in shaping the secondary immune response to the chimeric vaccine antigen H56, a promising vaccine candidate against *Mycobacterium tuberculosis*, consisting of the antigens Ag85B fused to the 6-kDa early secretory antigenic target and the latency-associated protein Rv2660c (25), already tested in four phase I and II clinical trials.^2^ The analysis of the serum IgG strength of binding to the vaccine antigen performed by surface plasmon resonance, and the computational analysis of the polyfunctional nature of reactivated CD4^+^ T cells, have been used to highligh the impact of the priming event in the induction of the adaptive immune response.

## Materials and Methods

### Mice

Seven-week-old female C57BL/6 mice, purchased from Charles River (Lecco, Italy), were housed under specific pathogen-free conditions in the animal facility of the Laboratory of Molecular Microbiology and Biotechnology, Department of Medical Biotechnologies at University of Siena. This study was carried out in accordance with national guidelines (Decreto Legislativo 26/2014). The protocol was approved by the Italian Ministry of Health (authorization no. 1004/2015-PR, 22 September 2015).

### Immunizations

**Figure 1:**
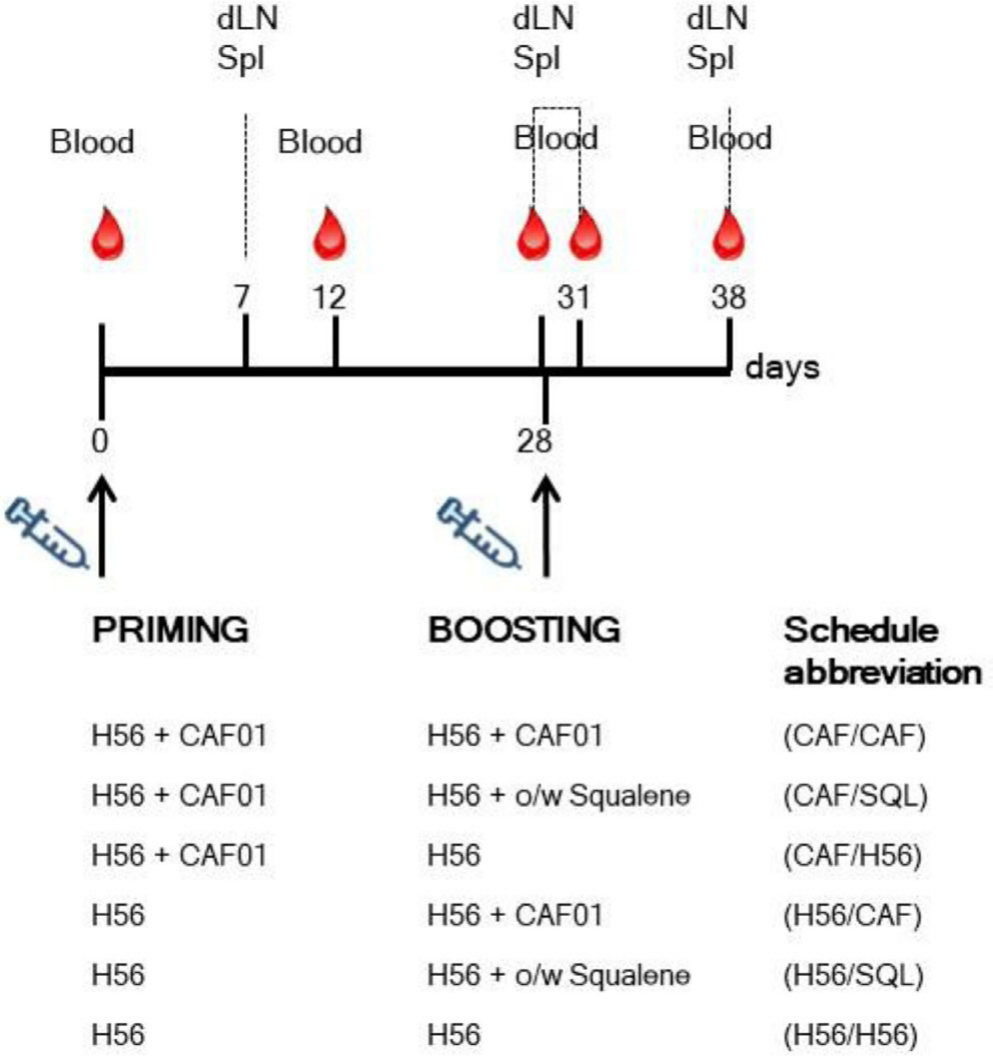
Study design and sample collection. Groups of C57BL/6 mice were subcutaneously primed with H56 alone (H56) or combined with CAF01, and boosted at day 28 with H56 + CAF01, or H56 + o/w squalene or H56 alone. Blood samples were collected at day 0, 12, 28, 31, and 38 following priming; draining lymph nodes (dLN) and spleens (Spl) were collected at day 7, 28, 31, and 38. The abbreviations of the different prime-boost combinations are here reported and used in all the figures.

Mice were immunized by the subcutaneous route at the base of the tail, with vaccine formulations including the chimeric tuberculosis vaccine antigen H56 (2 μg/mouse for priming, 0.5 μg/mouse for boosting; Statens Serum Institut, Denmark), administered alone or combined with the adjuvants CAF01 (250 µg dimethyldioctadecylammonium and 50 µg trehalose dibehenate/mouse; Statens Serum Institut, Denmark), or a squalene-based oil-in-water adjuvant [50 μl/mouse, sorbitan trioleate (0.5% w/v) in squalene oil (5% v/v), and Tween 80 (0.5% w/v) in sodium citrate buffer (10 mM, pH 6.5), Invivogen, USA]. Groups of five mice were primed with H56 or H56 + CAF01 and boosted with H56, H56 + CAF01, or H56 + o/w squalene, 4 weeks later, as reported in Figure 1. Formulations containing CAF01 were injected in a volume of 150 μl/mouse of Tris 10 mM, while formulations containing o/w squalene or H56 alone in a volume of 100 μl/mouse of PBS. Groups of mice were sacrificed 7 and 28 days after priming, and 3 or 10 days after boosting (Figure 1).

### Sample Collection and Cell Preparation

Blood samples were taken from the temporal plexus of mice 0, 12, and 28 days after priming and 3 and 10 days after boosting. Samples were incubated for 30 min at 37°C and centrifuged at 1,200 × *g* at 4°C for 10 min for collecting sera that were stored at −80°C. Draining lymph nodes (sub iliac, medial, and external) and spleens were collected 7 and 28 days after priming, and 3 or 10 days after boosting. Samples were mashed onto 70 µm nylon screens (Sefar Italia, Italy) and washed two times in complete RPMI medium [RPMI (Lonza, Belgium), 100 U/ml penicillin/streptomycin, and 10% fetal bovine serum (Gibco, USA)]. Samples were treated with red blood cells lysis buffer (eBioscience, USA) and counted with cell counter (Bio-Rad).

### Multiparametric Flow Cytometric Analysis

Samples from draining lymph nodes (dLN) and spleens were incubated for 30 min at 4°C in Fc-blocking solution (cRPMI with 5 µg/ml of CD16/CD32 mAb [clone 93; eBioscience, USA]). Cells from dLN were stained for 1 h at RT with PE-conjugated I-A(b) *M. tuberculosis* Ag85B precursor 280–294 (FQDAYNAAGGHNAVF) tetramer (kindly provided by NIH MHC Tetramer Core Facility, Emory University, Atlanta, GA, USA), washed and surface stained with HV500-conjugated anti-CD4 (clone RM4-5; BD Biosciences) and BV786-conjugated anti-CD44 (clone IM-7; BD Biosciences). Samples were labeled with Live/Dead Fixable Near-IR Stain Kit according to the manufacturer instruction (Invitrogen, USA). Intracellular cytokine production was assessed on splenocytes cultured for 6 h in the presence of anti-CD28, anti-CD49d (both 2 µg/ml, eBioscience), and H56 protein (2 µg/ml). Unstimulated or PMA and ionomycin calcium salt (50 ng/ml and 1 µM respectively, Sigma-Aldrich) treated cells were used as negative and positive controls, respectively. Brefeldin A (BFA, 5 µg/ml, Sigma-Aldrich) and monensin solution (1×, eBioscience) were added to all samples for the last 4 h of incubation. Cells were washed twice in PBS and labeled with Live/Dead Fixable Yellow Stain Kit according to the manufacturer instruction (Invitrogen, USA). Fixation and permeabilization were performed using BD Cytofix/Cytoperm kit according to the manufacturer instruction (BD Biosciences) before Fc-blocking and stained with HV500-conjugated anti-CD4 (clone RM4-5; BD Biosciences), BV786-conjugated anti-CD44 (clone IM-7; BD Biosciences), PerCP Cy5.5-conjugated anti-IFN-γ (clone XMG1.2; BD Biosciences), AF700-conjugated anti-TNF-α (clone MP6-XT22; BD Biosciences), APC-conjugated anti-IL-17A (clone eBio17B7; eBioscience), AF488-conjugated anti-IL-4 (clone 11B11; eBioscience), AF488-conjugated anti-IL-13 (clone eBio13A; eBioscience). All antibodies and tetramers were titrated for optimal dilution. About 5–10 × 10^5^ cells were stored for each sample, and acquired on BD LSRFortessa X20 flow cytometer (BD Biosciences). Data analysis was performed using FlowJo v10 (TreeStar, USA).

### Computation Analysis of Flow Cytometric Data with FlowSOM

Data from restimulated splenocytes were first analyzed with FlowJo. Live lymphocytes expressing CD4 and CD44 were manually gated, concatenated within the same immunization group, randomly downsampled to 15,000 cells and exported as uncompensated cells. Data were then compensated, logically transformed and scaled with FlowSOM (24). FlowSOM is a package of R, an open-source environment for statistical analysis, computation, and visualization,^3^ available as a Bioconductor package. Clustering analysis of data was performed following the FlowSOM function pipeline. The algorithm considers each cell in an *n*-dimensional space (where *n* = the number of cytokines considered for the analysis); cells with similar position in *n*-dimensional space are clustered together. After clustering, a SOM is built, where all clusters represent nodes and the nodes closely connected to each other resemble each other more than nodes that are only connected through a long path. The resulting clustering of the SOM is visualized in a minimal spanning tree. Three different sets of FCS files, named flowSet, were analyzed: (a) FCS files from the groups primed with H56 + CAF01 and boosted with H56, H56 + CAF01, and H56 + o/w squalene, (b) groups primed with H56 + CAF01 or H56 alone and boosted with H56 + CAF01, and (c) groups primed with H56 + CAF01 or H56 alone and boosted with H56 + o/w squalene. For each flowSet, a SOM (function “FlowSOM”) was built. Functions “CountGroups” and “PlotGroups” were used to visualize the flowSets as minimal spanning tree, to color the nodes depending on the expression of five cytokines and to highlight changes higher than twofold in each node.

### Enzyme-Linked Immunosorbent Assay (ELISA)

Serum H56-specific IgG were determined by ELISA. Flat bottomed Maxisorp microtiter plates (Nunc, Denmark) were coated with H56 (0.5 µg/ml) for 3 h at 37°C and overnight at 4°C in a volume of 100 µl/well. Plates were washed and blocked with 200 µl/well of PBS containing 1% BSA (Sigma-Aldrich) for 2 h at 37°C. Serum samples were added and titrated in twofold dilution in duplicate in PBS supplemented with 0.05% Tween 20 and 0.1% BSA (diluent buffer) in 100 µl/well. After 2 h at 37°C, samples were incubated with the alkaline phosphatase-conjugate goat anti-mouse IgG (diluted 1:1,000 in diluent buffer, Southern Biotechnology, USA) for 2 h at 37°C in 100 µl/well and developed by adding 1 mg/ml of alkaline phosphatase substrate (Sigma-Aldrich) in 200 µl/well. The optical density was recorded using Multiskan FC Microplate Photometer (Thermo Scientific). Antibody titers were expressed as the reciprocal of the highest serum dilution with an OD value ≥0.2, after subtraction of background values measured with diluent buffer alone.

### Binding of Anti-H56 Antibodies by Surface Plasmon Resonances

H56 antigen was immobilized on CM4 sensor chip (GE Healthcare) following standard amine coupling procedures. Antigen diluted at the concentration of 25 µg/ml in sodium acetate pH 3.5 was injected for 300 s at the flow rate of 5 µl/min over the sensor chip surface, previously activated with a 1:1 mixture of EDC-NHS. After immobilization, ethanolamine-HCl was injected for 7 min over all the surface to block any remaining active site on sensor chip surface. A blank immobilization was performed for the reference flow cell. Sera were diluted 200-fold in HBS-EP+ (10 mM Hepes, 150 mM NaCl, 3.4 M EDTA, 0.05% polysorbate 20, pH 7.4) and injected for 180 s at a flow rate of 30 µl/min onto immobilized H56, and dissociation phase was allowed for 300 s. Surface regeneration was achieved with a 45-s pulse of 10 mM glycine pH 2.0 at the same flow rate. For antibody isotyping, serum samples diluted 1:200 in HBS-EP+ were injected for 180 s across the H56-immobilized surface at 30 µl/min, allowing sample binding to the surface. Then anti-mouse IgM and anti-mouse IgG antibodies, respectively, diluted 1:250 and 1:500 in HBS-EP+ were sequentially injected for 120 s each at 30 µl/min. For anti-H56 monoclonal antibody binding, Hyb76-8 was diluted at different concentrations (40, 20, 10, 5, 2.5, 1, and 0.5 µg/ml) in HBS-EP+ and then injected for 180 s at the flow rate of 30 µl/min onto immobilized H56, and dissociation phase was allowed for 450 s. Surface regeneration was achieved with a 30-s pulse of 10 mM glycine pH 2.0 at 30 µl/min.

The experiments were performed on a Biacore T100 instrument (GE Healthcare). The actual binding response of samples (RU) was obtained by subtracting the background response, recorded by injecting the sample through the reference flow cell. Kinetic of anti-H56 mAb was analyzed with the “Biacore T100 evaluation 1.1.1” software using the 1:1 Langmuir model for fitting the curves. Dissociation rates were calculated by curve-fitting analysis to a dissociation model.

### Statistical Analysis

Mann–Whitney test for multiple pairwise comparisons was used for assessing statistical difference between groups receiving the same booster and primed with H56 alone or H56 + CAF01 adjuvant. Kruskal–Wallis test, followed by Dunn’s post test for multiple comparison, was used to assess statistical difference among all groups. IgG titers were reported as geometric mean titers (GMT) with 95% CI, and statistical analysis was performed on log-transformed data. Statistical significance was defined as *P* ≤ 0.05. Analysis was performed using GraphPad Prism v7 (GraphPad Software, USA).

## Results

### Primary Ag-Specific CD4^+^ T Cell Expansion and Effector Function

**Figure 2:**
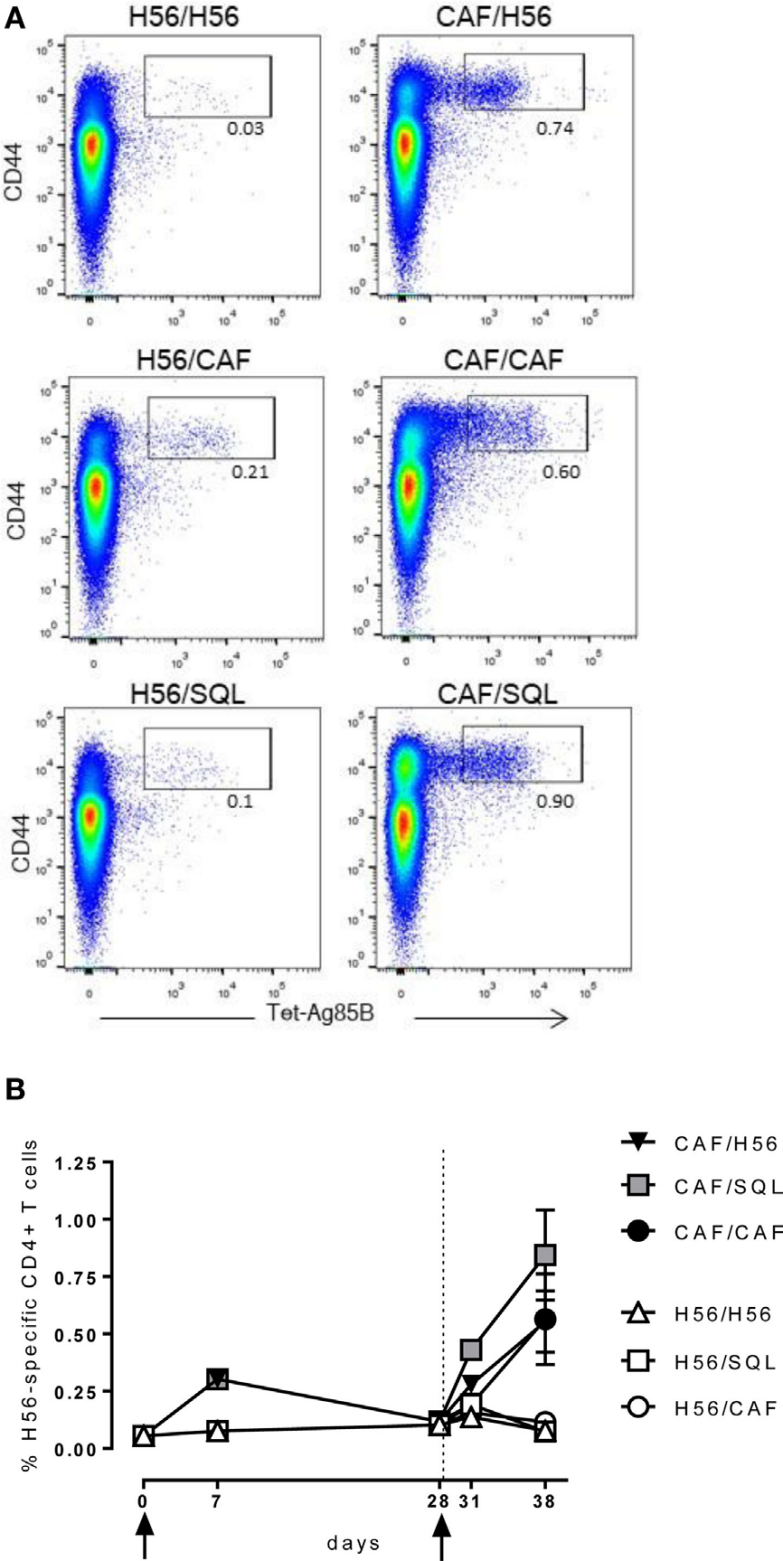
Ag85B-specific CD4^+^ T cell response. C57BL/6 mice were subcutaneously immunized as summarized in Figure 1. Draining lymph nodes were collected 7 and 28 days after priming, and 3 and 10 days after booster immunization and analyzed for the frequency of Ag-specific CD4^+^ T cells, identified by staining with Ag85B-specific MHC class II tetramers (Tet-Ag85B). **(A)** Scatter plot of CD44 versus Tet-Ag85B, gated on live CD4^+^ lymphocytes, shown from a single animal representative of each immunization group, collected 10 days after boosting. Activated tetramer^+^ cells are gated and the frequency reported. **(B)** Time course analysis of the frequencies of tetramer^+^ CD4^+^ T cells, detected in each group, reported as mean ± SEM of five mice per group. Mann–Whitney test for multiple pairwise comparisons was used for assessing statistical difference between groups receiving the same booster and primed with H56 alone or H56 + CAF01 adjuvant. Kruskal–Wallis test, followed by Dunn’s post test for multiple comparison, was used to assess statistical difference among all groups (*P* ≤ 0.05).

Groups of mice were parenterally primed with the chimeric tuberculosis vaccine antigen H56 administered alone or combined with the liposome system CAF01. Four weeks later, mice were boosted with three different formulations: the H56 antigen alone, the H56 antigen combined with CAF01, or mixed with an oil-in-water (o/w) squalene-based emulsion, hereafter o/w squalene (Figure 1). The induction of antigen-specific CD4^+^ T cell expansion into the draining iliac lymph nodes was assessed 7 and 28 days after priming, and 3 and 10 days after booster immunization. Antigen-specific CD4^+^ T cells were identified using Ag85B_280–294_-complexed MHC class II tetramers, specific for the immunodominant epitope of Ag85B, that is part of the chimeric H56 protein. Staining specificity was determined using a control tetramer complexed with an unrelated antigen that showed a level of staining below 0.02% (data not shown). Representative dot plots showing the frequencies of tetramer-positive (Tet^+^) T cells elicited by the different vaccine formulations 10 days after boosting are shown in Figure 2A. As clearly observed, all the groups primed with the vaccine formulation containing the CAF01 adjuvant, developed a recall response of the CD4^+^ T cells significantly higher compared to groups primed with H56 antigen alone (Figure 2B, *P* ≤ 0.05). The frequency of antigen-specific CD4^+^ T cells was higher in mice primed with H56 + CAF01 and boosted with H56 + o/w squalene versus all groups primed with H56 (*P* ≤ 0.05) while no significant differences were observed versus groups boosted with H56 alone or H56 + CAF01 (CAF/H56 and CAF/CAF). The secondary response clearly reflects the reactivation of cells activated by the primary immunization with H56 + CAF01, observed 7 days after priming (Figure 2B).

**Figure 3:**
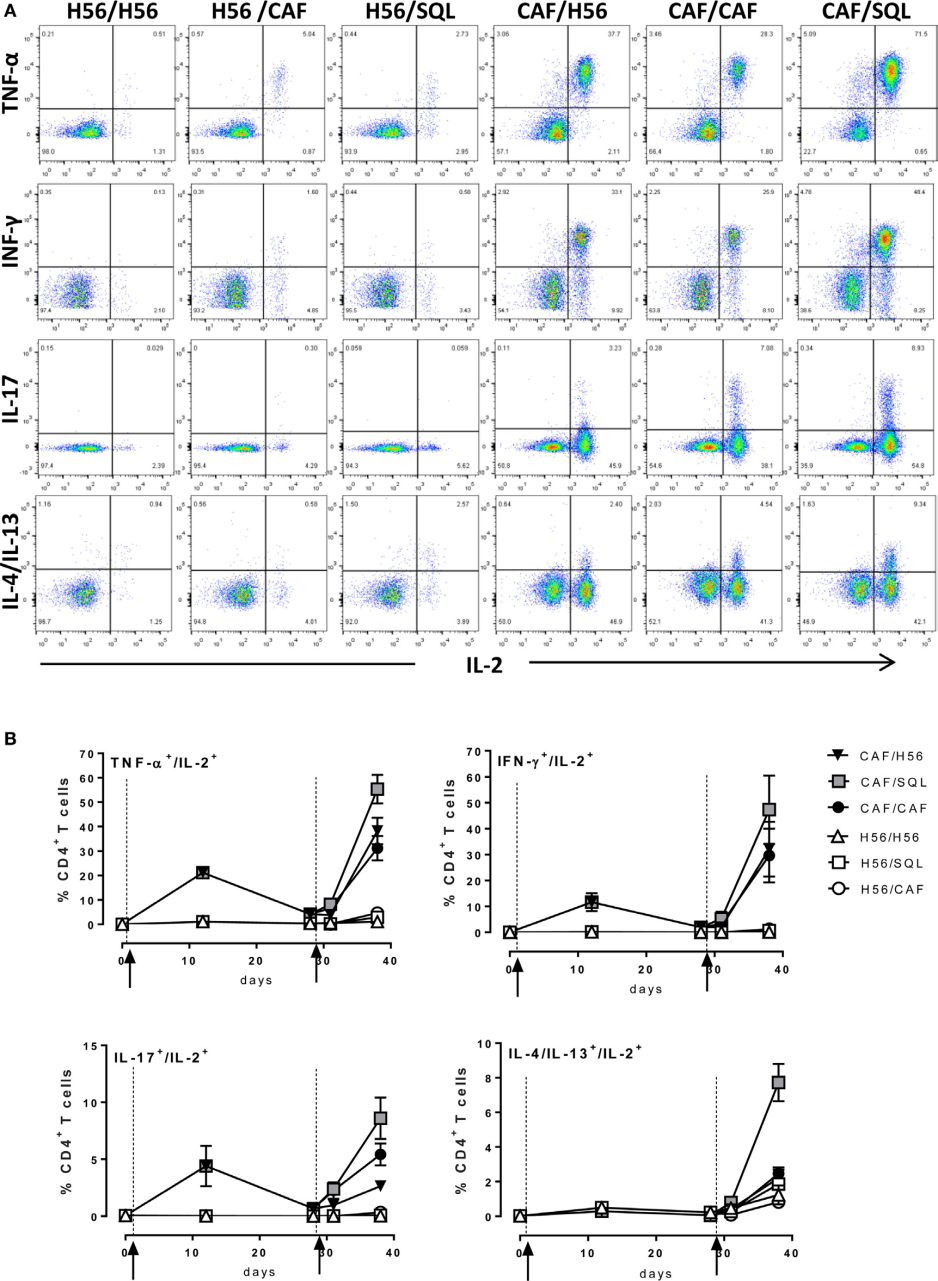
Intracellular cytokines production. C57BL/6 mice were subcutaneously immunized as summarized in Figure 1. Spleens were collected 7 and 28 days after priming, and 3 and 10 days after boosting. Splenocytes were restimulated for 6 h with H56 protein. **(A)** Dot plots showing the production of TNF-α, IFN-γ, IL-17, IL-4/IL-13 versus IL-2 assessed on live CD4^+^ CD44^+^ lymphocytes in each group, collected 10 days after boosting. **(B)** Percentages of T cells positive for both IL-2 and the indicated cytokines, with respect to total CD4^+^ CD44^+^ cells, elicited by different vaccine formulations reported as mean ± SEM of five mice per group. Mann–Whitney test for multiple pairwise comparisons was used for assessing statistical difference between groups receiving the same booster and primed with H56 alone or H56 + CAF01 adjuvant. Kruskal–Wallis test, followed by Dunn’s post test for multiple comparison, was used to assess statistical difference among all groups (*P* ≤ 0.05).

Since most of T helper cells activated into the draining lymph nodes exit and recirculate, the effector function of H56-specific CD4^+^ T cells was assessed in the spleen, analyzing the intracellular production of different cytokines using multicolor flow cytometry. Dot plots, representative of the different groups, showing the frequencies of H56-specific CD4^+^ T cells producing TNF-α, IFN-γ, IL-17, or IL-4/IL-13 cytokines versus IL-2, a cytokine indicative of the proliferative response and activation program of antigen-specific T cells, are reported in Figure 3A. The analysis of the effector function clearly confirmed the importance of the priming event to elicit cells capable of reactivation upon antigen restimulation. Indeed, only groups of mice that had been primed with H56 + CAF01 were able to reactivate, upon boosting, cells co-expressing IL-2 with TNF-a (55%, 38%, and 31% in CAF/SQL, CAF/H56, and CAF/CAF groups, respectively), with IFN-γ (47%, 32%, and 30%, respectively) or with IL-17 (9%, 3%, and 6%, respectively), with percentages of effector cells significantly higher compared to the respective groups primed with H56 (Figure 3B; Mann–Whitney test for multiple pairwise comparisons, *P* ≤ 0.05). Interestingly, the booster immunization with o/w squalene, an adjuvant capable of stimulating a primary Th1 and Th2 mixed response (4), increased the frequency of cells releasing IL-2 with IL4 and IL13 (7%; *P* ≤ 0.05 versus the respective group H56/SQL), even though these cytokines were not observed following priming with H56 + CAF01 (Figure 3B). It was also clearly shown that mice primed with H56 alone did not respond to the booster immunization, also when CAF01 or o/w squalene adjuvants were used (Figure 3B).

### Computational Analysis of Intracellular Cytokines Production

**Figure 4:**
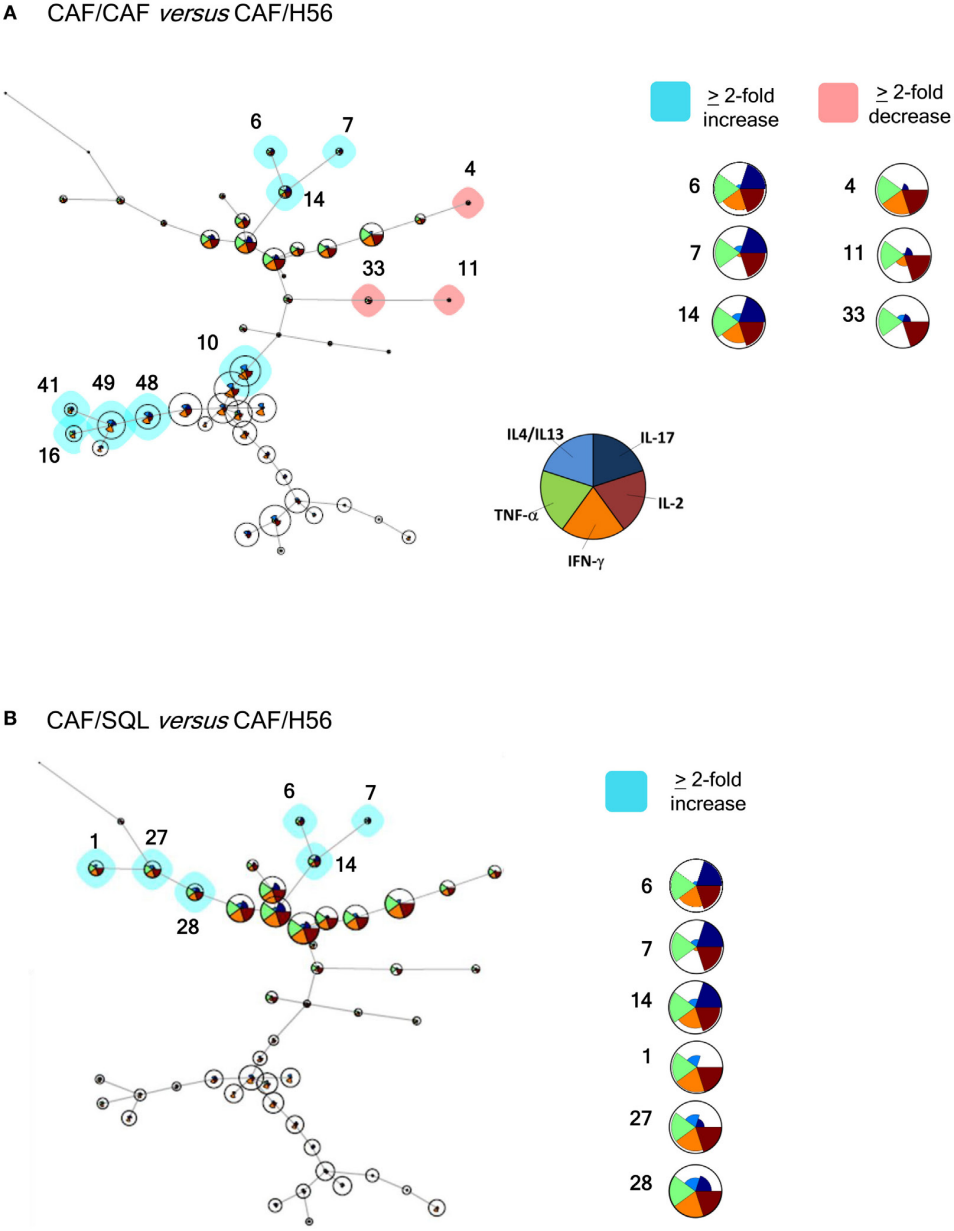
Computational analysis of polyfunctional profiles of T cells. C57BL/6 mice were subcutaneously immunized as summarized in Figure 1. CD4^+^ and CD44^+^ cells from restimulated splenocytes were manually gated with FlowJov10, concatenated within the same immunization group, randomly downsampled to 15,000 cells and exported as uncompensated cells. Data were then compensated, logically transformed, and scaled with FlowSOM. A minimal spanning tree was obtained from the flowSet including FCS files of the groups primed with H56 + CAF01 and boosted with H56, H56 + CAF01, and H56 + o/w squalene. Size of the nodes is relative to the percentage of cells present in each cluster. Each node is represented by a star chart indicating the relative mean fluorescence intensity values of each cytokine; the height of each sector indicates the intensity, if the part reaches the border of the circle, the cells have maximal expression for that cytokine. A relative increase (blue nodes) or decrease (pink nodes) of at least twofold of the cell percentage in nodes of the CAF/CAF **(A)** or CAF/SQL **(B)** groups compared with the CAF/H56 group is shown. A magnification or colored nodes, indicated by numbers, is reported on the right.

In order to have a global picture of the polyfunctional profiles of T cells elicited by the different vaccine formulations, a computational analysis of data was performed using the multidimensional software FlowSOM. A minimal spanning tree was built connecting the nodes that were most similar to each other in minimal branches. Each cell was then classified to the nearest node that was coded as a pie chart with information about the expression of the five different cytokines (Figure 4). The mean fluorescence intensity (MFI) values of each cytokine are visualized inside each node. The height of each sector indicates the intensity, therefore when it reaches the border of the circle, this indicates that these cells have a high expression for that cytokine. The co-expression intensity of more cytokines by a group of cells clustered into the same node can be easily visualized. Moreover, the size of the nodes indicates the number of cells assigned to each node, so it is indicative of the frequency of activated CD4^+^ T cells expressing that pattern of cytokines.

**Figure 5:**
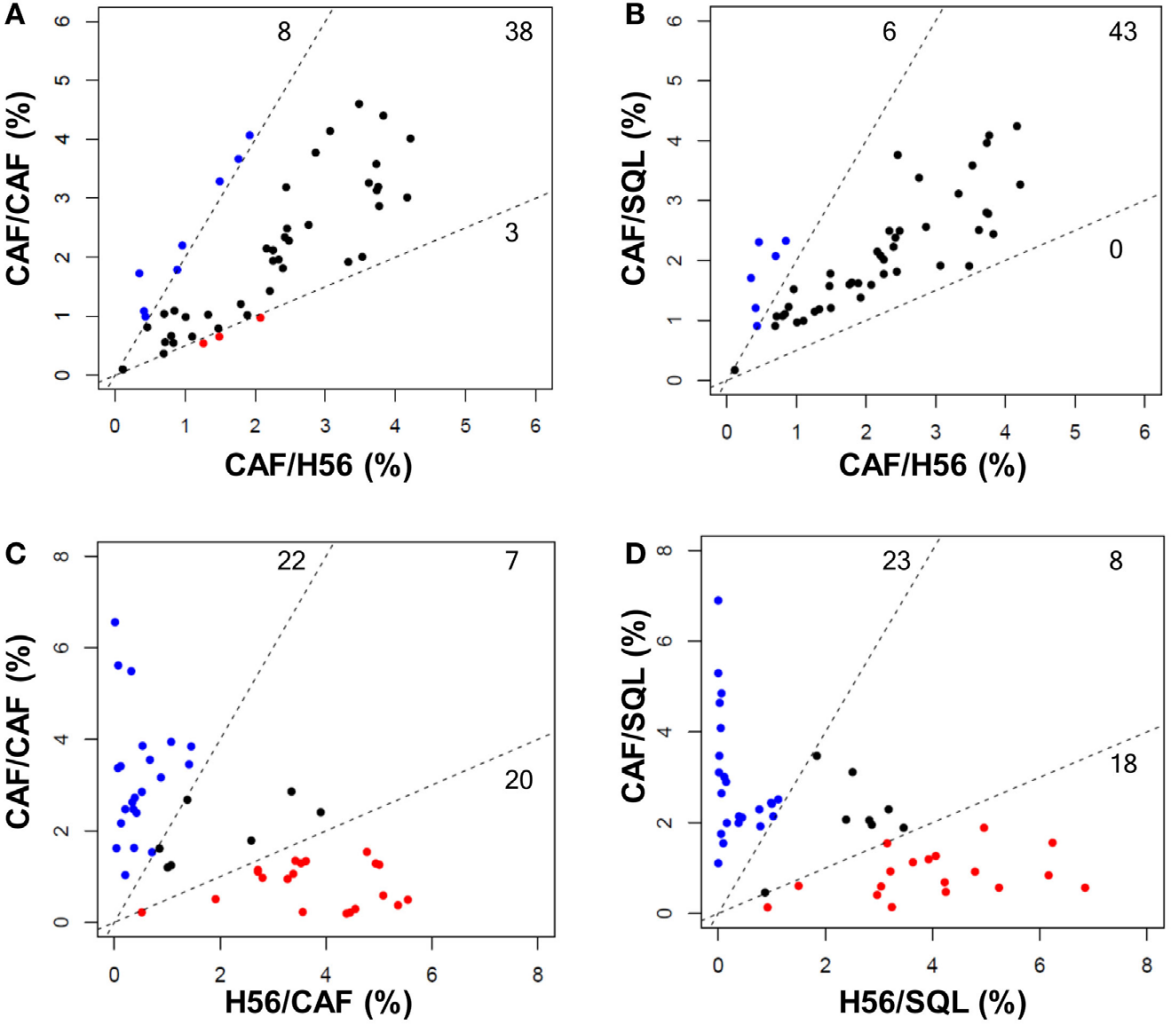
Comparison of FlowSOM clustering in different prime-boost combinations. Scatter plots showing the frequency of cells within the 49 nodes obtained with the FlowSOM analysis. Groups with the same priming and different boost are shown in **(A,B)** (CAF/CAF versus CAF/H56, CAF/SQL versus CAF/H56, respectively); groups with the same boost and different priming are shown in **(C,D)** (CAF/CAF versus H56/CAF and CAF/SQL versus H56/SQL, respectively). Each dot represents a node, and the *X* and *Y* axis report the percentage of cells clustered into this node calculated in the indicated prime-boost combination. Within the dashed lines are reported nodes (black) with a similar frequency of cells assessed in the two immunization combinations; nodes with a frequency of cells >2-fold with respect to the other immunization group are reported in blue or red. The threshold value was chosen according to default threshold in FlowSOM package. The number of nodes is reported in each sector.

Trees obtained in the groups primed with H56 + CAF01 and boosted with H56 + CAF01 (CAF/CAF) or H56 + o/w squalene (CAF/SQL) are shown in Figures 4A,B, respectively. Forty nine nodes were created by the FlowSOM algorithm. The upper half of the trees shows nodes co-expressing more cytokines, in particular TNF-α, IL-2, and IFN-γ, with a high MFI. On the contrary, cells expressing very few cytokines with low MFI are shown in the lower half of the tree. When the frequency of cells within a node increased or decreased by at least twofold with respect to mice boosted with H56 alone, nodes were colored in blue or pink, respectively (Figures 4A,B). Mice boosted with CAF01 increased the frequency of cells expressing the pattern of cytokines shown in nodes 6, 7, 14 (upper half of the tree), and 10, 16, 41, 48 and 49 (lower half of the tree). Nodes 6, 7, and 14 include cells with a high expression of IL-17 together with TNF- α, IL-2 (node 7), and also IFN-γ (nodes 6 and 14) as shown in the magnification reported on the right of Figure 4A. The other nodes (10, 16, 41, 48, and 49) include cells with a weak expression of some cytokines, as indicated by the height of the sectors. At the same time, CAF01 induced the reduction of nodes 4, 11, and 33 (pink), which express TNF-α, IL-2 (nodes 11 and 33), and also IFN-γ (node 4). Interestingly, the cluster of cells co-expressing IL-17 (nodes 6, 7, and 14) was significantly increased also with o/w squalene with respect to H56 alone (Figure 4B). The amount of cells co-expressing TNF- α, IL-2, IFN-γ with an intermediate MFI of IL-4/IL-13 (nodes 1, 27, and 28) was also increased (Figure 4B). The frequency of cells clustered within single nodes for each immunization schedule is reported in Figure 5. Here, we can observe how many nodes the two plotted groups have in common, with a similar percentage of cells clustered inside (black dots), and how many nodes include an amount of cells ≥2-fold with respect to the compared immunization group (colored dots). In Figure 5 panels A and B, the comparison of FlowSOM clustering between groups primed with H56 + CAF01 and boosted with adjuvanted formulations or H56 alone is reported, reflecting the differences shown in Figure 4. Panels C and D show the comparison between groups receiving different priming (H56 alone or with CAF01) but boosted with the same formulation (either H56 + CAF or H56 + o/w squalene). In this case, the amount of common nodes between the compared formulations drastically diminished while more than 40 nodes were differently clustered among compared groups (Figures 5C,D).

As a whole, we can conclude that there are some clusters of cells, mainly oriented toward the secretion of IL-17 together with other cytokines, that significantly increase due to the presence of the adjuvants in the booster immunization, while the rest of the nodes do not significantly change between different boosting formulations. On the contrary, a drastical change of most of the nodes is observed comparing groups receiving a priming with or without the adjuvant, confirming again that the priming setting defines the generation of cells capable of secondary reactivation. The overall CD4^+^ T cell response analysis clearly underlines the different roles of the priming and boosting events with respect to the adaptive immune response.

### Humoral Immune Response

**Figure 6:**
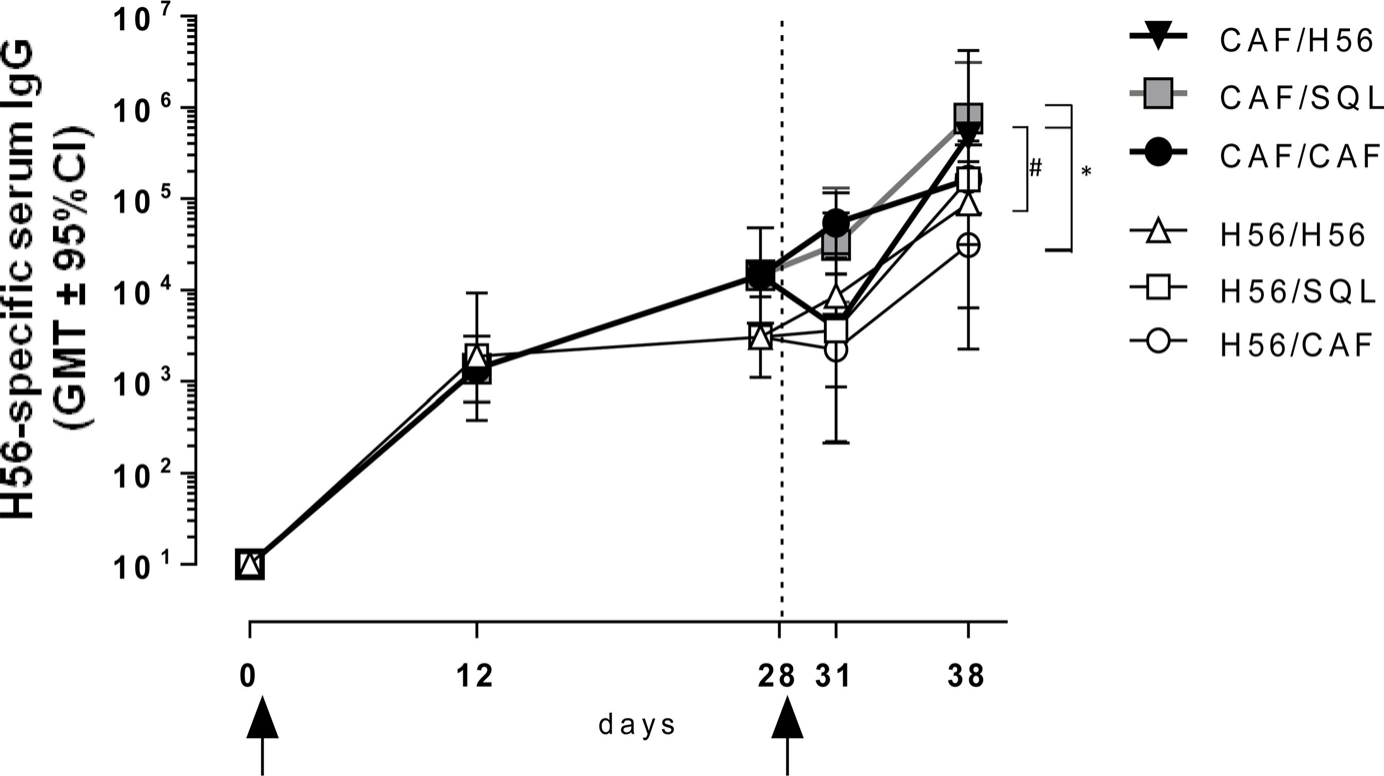
Antigen-specific IgG response. C57BL/6 mice were subcutaneously immunized as summarized in Figure 1. H56-specific IgG serum titers were analyzed on day 0, 12, 28, 31, and 38 following priming, by enzyme-linked immunosorbent assay. Antibody titers are expressed as the reciprocal of the highest serum dilution with an OD value ≥0.2 after subtraction of background value (diluent buffer). Values are reported as geometric mean titers (GMT) ± 95% CI of 8–10 mice per group from two independent experiments. Mann-Whitney test for multiple pairwise comparisons was used for assessing statistical difference between groups receiving the same booster and primed with H56 alone or H56 + CAF01 adjuvant, ^#^*P* ≤ 0.05. Kruskal–Wallis test, followed by Dunn’s post test for multiple comparison, was used to assess statistical difference between all groups. *P ≤ 0.05.

The induction of antigen-specific IgG antibody response was assessed at different time points after priming and boosting (Figure 1). The primary response elicited by both H56 alone or combined with CAF01 was very similar at 12 and 28 days after priming, while the secondary IgG response showed significant differences according to the formulation used for the booster immunization (Figure 6). Mice primed with H56 + CAF01 and boosted with H56 + o/w squalene, or with H56 alone developed the highest humoral response compared to all the other groups (*P* ≤ 0.05 of CAF/H56 versus H56/CAF). A significant difference was also detected between the two groups boosted with H56 alone but differently primed (*P* ≤ 0.05), indicating that a consistent recall antibody response can be elicited also by the antigen alone when an efficient priming had been performed.

**Figure 7:**
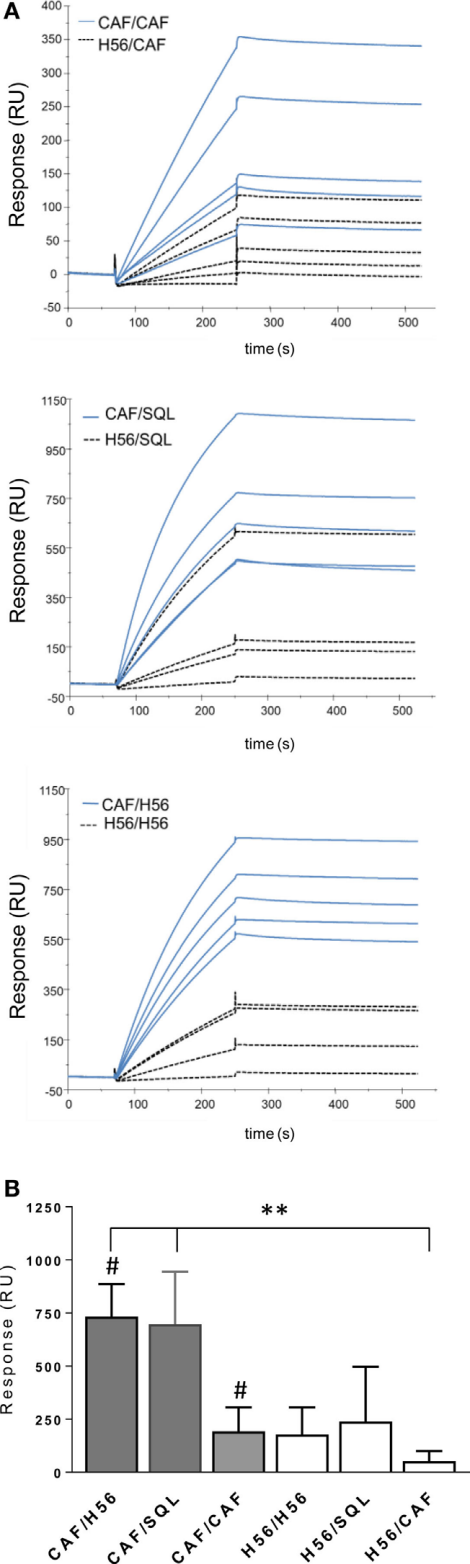
Surface plasmon resonance analysis of H56-specific sera. A Biacore assay was performed on sera collected 10 days after booster immunization. Sera were diluted 200-fold and injected over H56 previously immobilized on CM4 sensor chip. **(A)** Sensorgrams of H56-specific antibodies binding (RU) from sera of single animals primed with the CAF01 adjuvant (blue lines) or with H6 alone (dashed lines). **(B)** Mean RU values ± SEM of five sera for group; filled bars indicate groups primed with CAF01 adjuvant, open bars with antigen alone. Mann–Whitney test for multiple pairwise comparisons was used for assessing statistical difference between groups receiving the same booster and primed with H56 alone or H56 + CAF01 adjuvant, ^#^*P* ≤ 0.05. Kruskal–Wallis test, followed by Dunn’s post test for multiple comparison, was used to assess statistical difference between all groups. **P ≤ 0.01.

Using surface plasmon resonance, we further compared sera collected after boosting for the capacity of binding to H56 immobilized on the sensor chip surface and data were expressed as resonance units (RU). The antibody isotype characterization indicated a complete absence of IgM in all the analyzed samples, thus allowing to correlate RU with IgG concentration (Figure S1 in Supplementary Material). Sensorgrams from single animals primed with or without the adjuvant and boosted with the same vaccine formulations showed higher binding responses to H56 in groups that had received a primary immunization with CAF01, compared to sera from mice primed with H56 alone (Figure 7A). The mean values of RU calculated for each immunization group are reported in Figure 7B. H56-specific IgG binding response in groups primed with H56 + CAF01 and boosted with H56 + o/w squalene or with H56 alone was of 732 and 700 RU respectively, while the group boosted with CAF01 showed a lower RU (190), even if it was significantly higher compared to the corresponding group primed with H56 alone (*P* ≤ 0.05). The dissociation kinetic rates (koff) calculated for each curve did not show significant differences among the differently immunized groups (Figure S2B in Supplementary Material) and were similar to the koff value of a monoclonal anti-H56 antibody (Hyb76-8) used as reference (Figure S2A in Supplementary Material).

The analysis of the humoral response again confirms the importance of the correct combination of vaccine formulations to be used for priming and boosting, and strengthens the importance of the presence of the CAF01 adjuvant in the formulation used for priming rather than for boosting, in line with the cellular data.

## Discussion

Heterologous prime-boost combinations including two different vaccine adjuvants were used to study the role of the CAF01 adjuvant in generating primary immune response. The analysis of the secondary antigen-specific immune response upon booster immunization has been instrumental for evaluating (i) the impact of the priming and boosting events on the immune response to vaccination, (ii) the role of the adjuvant component in the primary and booster immunization, and (iii) the impact of the vaccine formulation on the functional profile of the cellular response elicited.

The prime-boost study reported here demonstrates that a primary immunization performed with the vaccine antigen and the CAF01 adjuvant deeply impacts the immune response of the host, generating T and B cells capable of responding to recall immunization also when it is performed with the vaccine antigen alone. On the contrary, when the H56 vaccine antigen alone is used for priming, the secondary T immune response is completely abolished by the booster immunization, even in the presence of formulations including two potent adjuvants, such as CAF01 or o/w squalene, and the serum humoral response is much lower. The properties of these two adjuvants in eliciting an H56-specific primary immune response, have been previously characterized by our group (4). We demonstrated that the CAF01 adjuvant promotes a Th1 and Th17 primary immune response, stimulates the GC reaction inside the draining lymph nodes, and promotes a slower response in terms of early serum antibodies compared to other adjuvants, probably due to its mechanism of action that entraps the antigen slowing down its release. The o/w squalene adjuvant stimulates a primary cellular response characterized by the release of TNF-α, IFN-γ, and IL-4/IL-13 indicative of a mixed Th1/Th2 response, and elicits a rapid and significant humoral response in serum (4). The mixed combination of CAF01 for priming and o/w squalene for boosting tested here was extremely immunogenic both in terms of cellular and humoral response. A reactivation of the H56-specfic T helper response was elicited into the draining lymph nodes, and the effector function of reactivated cells reflected the profile elicited by the priming event, as shown by the production of TNF-α, IFN-γ, IL-2, and IL-17. Nevertheless, at the same time, there was a significant increase also of cells producing the cytokines IL-4 and IL-13, that were not elicited by the priming with CAF01, but were observed in mice primed with o/w squalene adjuvant (4). As shown by the computational analysis of cytokine production, cells expressing IL-4/IL-13 produced also TNF-α, IFN-γ, and IL-2. The analysis of the secondary immune response elicited in the group primed with H56 + CAF01 demonstrated that the use of the antigen alone for booster immunization was extremely efficient. In this case, there was a significant reactivation of the T helper response, also in terms of effector cells. We observed that antigen alone stimulates the reactivation of cells releasing TNF-α, IFN-γ, and IL-2, but not high levels of IL-17, that was observed only in groups boosted with each of the adjuvanted formulations.

The induction of cells secreting these cytokines is critical in the host immune response to *M. tuberculosis*. IFN-γ is crucial for the activation of macrophages, which in turn inhibit *M. tuberculosis* growth *via* induction of inducible isoform of nitric oxide synthase and autophagy (26), while TNF-α promotes the formation of mature granulomas and also activates infected macrophages. IL-2 stimulates the expansion and maintenance of the T cell responses, therefore contributes to the host defense, and loss of IL-2-producing CD4^+^ T cells is associated with loss of protection (27). The requirement for cells producing IL-17 to control the pathogen is less absolute, even if vaccine promoted Th17 cells can improve mycobacterial control in animal models, promoting early Th1 cell recruitment to the lung following aerosol *M. tuberculosis* infection and reduce bacterial burden (28, 29).

The H56-specific humoral response was induced by formulations containing the antigen alone or combined with adjuvants, with higher antibody titers observed in mice primed with H56 + CAF01 and boosted with H56 + o/w Squalene, or with H56 alone. The latter formulations also elicited antibodies with the highest binding properties for the vaccine antigen. The analysis performed in the groups primed with H56 alone showed that also in the presence of adjuvants in the booster immunization, the secondary antibody response was lower, with a very low binding capacity to the vaccine antigen. Previous studies have shown a complete absence of GC B and follicular T helper cells when the primary immunization was performed with antigen alone, while short-lived plasma cells were induced (4). Interaction of Tfh cells with B cells drives the GC reaction, a dynamic micro-anatomical structure that supports the generation of B-cell activation, antibody class switch recombination, and affinity maturation (30, 31). The lack of the GC reaction, together with the induction of short-lived plasma cells capable of secreting low affinity antibodies (32), can explain the quality and the quantity of the humoral secondary response observed in mice primed with H56 alone. To note, a completely opposite result in terms of vaccine antigen binding capacity was obtained in mice primed with H56 and boosted with the H56 + CAF01, versus mice primed with the H56 + CAF01 and then boosted with the H56 alone.

The strong difference between the impact of the priming and boosting event on the immune response was clearly visualized with the computational analysis of the cytokine profile of reactivated CD4^+^ T cells. The use of software capable of managing the huge amount of data produced by flow cytometry for each cell has become a necessity. Multiparametric data can no longer be adequately analyzed using the classical, mostly manual, analysis techniques, in which two parameters are combined in two-dimensional scatterplots, and therefore require the use of novel computational techniques (23). Among many softwares now available (33), we employed the FlowSOM software (9), which is a platform of analysis available as an open-source package for R, an open-source environment for statistical analysis, computation, and visualization. A SOM is an unsupervised technique for clustering and dimensionality reduction, in which a discretized representation of the input space is trained. The graphical output generated is helpful for visually displaying T cell polyfunctionality. This analysis clearly allowed to visualize a clusterization of cells producing different patterns of cytokines and to compare the polyfunctional activity of CD4^+^ T cells elicited by the different prime-boost combinations. Six different clusters were observed among groups primed with the CAF01 adjuvant and boosted with the same formulation, or with the o/w squalene adjuvant or with the vaccine antigen alone. Both adjuvants used for boosting increased the amount of cells producing IL-17 together with TNF-α, IL-2, and IFN-γ, while only o/w squalene increased the frequency of cells co-expressing also IL-4 and IL-13. This analysis highlighted that also in the presence of the antigen alone we could reactivate a polyfunctional response, but the use of the adjuvant can be instrumental for modulating a specific type of effector cells. The number of clusters significantly different between groups primed with or without the CAF01 adjuvant, and boosted with the same formulation increased to 42, confirming again that the priming formulation defines the generation of cells capable of secondary reactivation.

The role of vaccine-induced polyfunctional CD4^+^ T cells in the protection from *M. tuberculosis* infection is not completely clear, and results obtained in preclinical and clinical studies are sometimes contradictory (34). In the mouse model, the magnitude of polyfunctional CD4^+^ T cells often correlates with vaccine-induced protection, generally assessed as vaccine-induced control of bacterial replication following challenge, thus making polyfunctional T cells a good candidate for a mechanistic correlate of protection (34). It has been demonstrated that immunity elicited by H56 + CAF01 vaccination is associated with the maintenance of circulating polyfunctional CD4 T cells, that selectively home to the lung parenchyma, and confer durable protection (35). It is likely that cells expressing multiple effector functions may be more effective in controlling infection than those producing a single pro-inflammatory cytokine. Nevertheless, in humans, same cases have been reported in which there was a progress to disease, also in the presence of a strong Th1 responses (25, 28), and very little data on the role of IL-17 are available (36). Taken together, polyfunctional CD4^+^ T cells deserve to be assessed and characterized, but other functional immunological components, including the humoral responses (37) should be analyzed and considered when assessing vaccine candidates to *M. tuberculosis* (38).

The data from the current study in mice emphasizes the role of adjuvant for the priming of an optimal immune response, whereas the adjuvant seems to be of less importance during the boosting. In particular, CAF01 seems to have the ability to prime a response that results in a qualitatively and quantitatively superior antibody response. Ongoing clinical trials with CAF01 adjuvanted priming vaccines will soon demonstrate if these data can be translated into humans. If we in the future should implement heterologous immunization protocols in humans will eventually rely not only on immunogenicity but also on a number of practical considerations of great importance for vaccine implementation.

In conclusion, the primary stimulation of the immune system is crucial for the generation of cells capable of a recall response. The choice of an immunogenic vaccine formulation for priming event paves the path for the subsequent secondary response, while the choice of the formulation for boosting can be a tool for modulating the quality, more than the quantity, of the secondary response. The heterologous prime-boost approach for vaccination appears as an excellent strategy for the generation of vaccines specifically designed for specific pathogens.

## Ethics Statement

This study was carried out in accordance with national guidelines (Decreto Legislativo 26/2014). The protocol was approved by the Italian Ministry of Health (authorization no. 1004/2015-PR, 22 September 2015).

## Author Contributions

AC, EP, GPo, and DM contributed conception and design of the study; AC, EP, FF, and GPa performed the experiments; AC, EP, FF, and DM analyzed the data; SL, FS and AC performed computational analysis of flow cytometric data; JB performed and analyzed surface plasmon resonance data; PA provided reagents; AC wrote the manuscript; EP, FF, and JB wrote sections of the manuscript; GPo, DM, PA and LB critically revised the manuscript. All authors contributed to manuscript revision, read, and approved the submitted version.

## Conflict of Interest Statement

PA is a co-inventor on patent applications covering CAF01. As an employee, PA has assigned all rights to Statens Serum Institut, a Danish non-profit governmental institute. All other authors that the research was conducted in the absence of any commercial or financial relationships that could be construed as a potential conflict of interest.

## Footnotes

^1^www.iprove-roadmap.eu.

^2^http://ClinicalTrials.gov.

^3^www.r-project.org.
