# Supplementary material for: Heterologous Prime-Boost Combinations Highlight the Crucial Role of Adjuvant in Priming the Immune System
Source: Front Immunol. 2018 Mar 12;9:380. doi: 10.3389/fimmu.2018.00380 (PMC5857569; doi:10.3389/fimmu.2018.00380)
Supplement: Supplementary file 1 [file image_1.PDF]

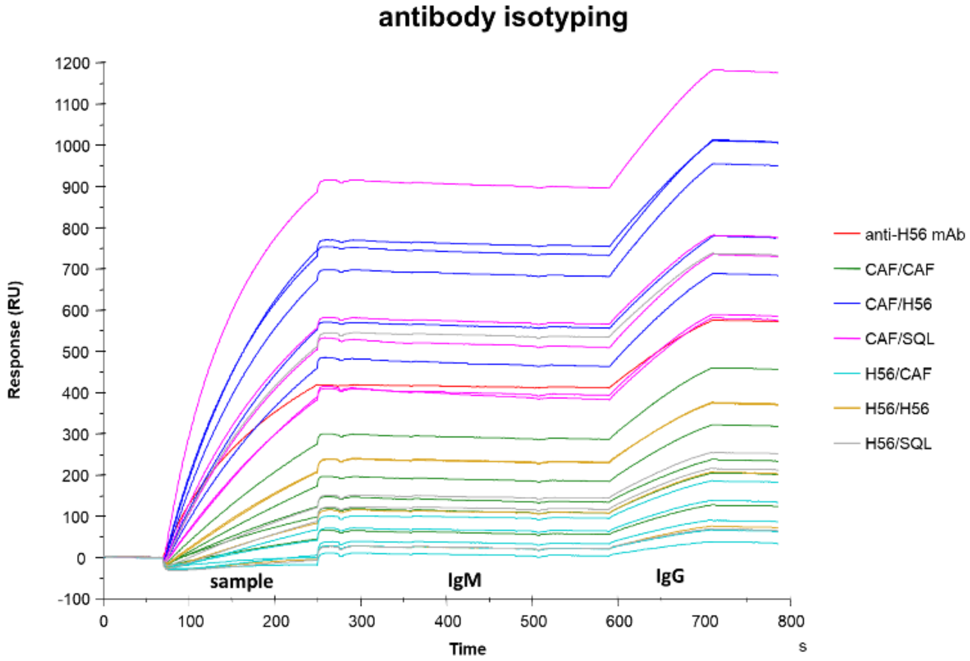

Figure S1. Surface plasmon resonance analysis of antibody isotype in immunized sera Characterization of the antibody isotype in each serum sample collected 10 days after booster immunization. Antibody isotyping was performed by sequential injections of anti-mouse IgM and IgG antibodies following the injection of each serum sample. Colors indicate animals in the different immunized groups.
