# Supplementary material for: Heterologous Prime-Boost Combinations Highlight the Crucial Role of Adjuvant in Priming the Immune System
Source: Front Immunol. 2018 Mar 12;9:380. doi: 10.3389/fimmu.2018.00380 (PMC5857569; doi:10.3389/fimmu.2018.00380)
Supplement: Supplementary file 2 [file image_2.PDF]

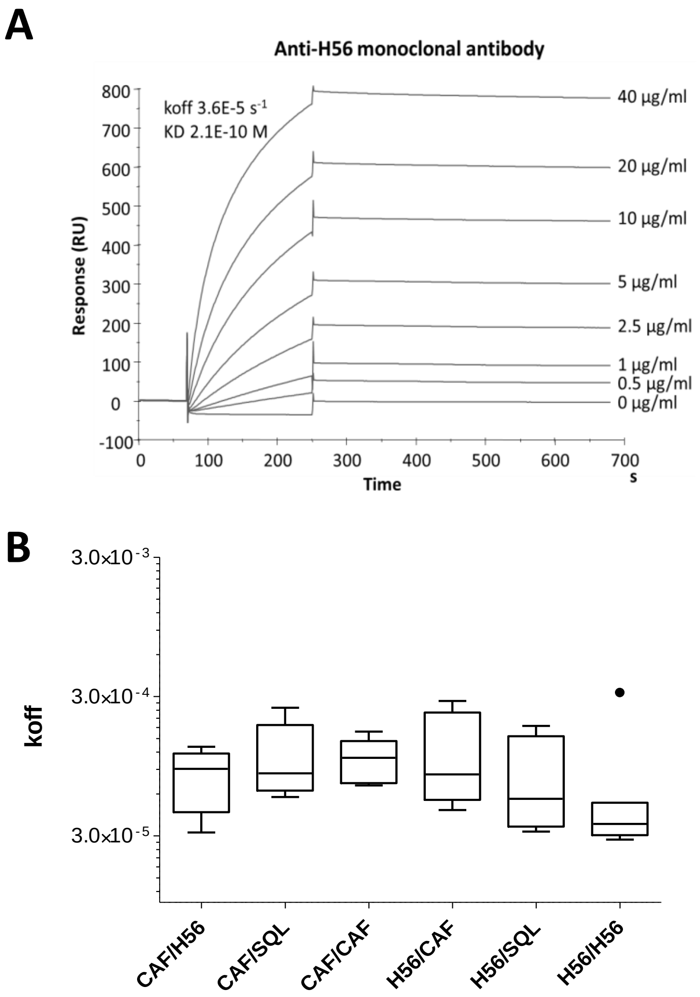

Figure S2. Surface plasmon resonance analysis of monoclonal antibody Hyb76-8 and of H56-specific sera. A. Sensorgram of Hyb76-8 monoclonal antibody (mAb) diluted at different concentrations (40, 20, 10, 5, 2.5, 1 and 0.5 µg/ml) and then injected onto immobilized H56. Kinetic of mAb was analyzed with the "Biacore T100 evaluation 1.1.1" software using the 1:1 Langmuir model for fitting the curves. **B**. Koff values determined by SPR analysis of sera collected 10 days after booster immunization. Bars represent mean values ± SD of 5 samples for each immunization group.
